# Measuring Independence between Statistical Randomness Tests by Mutual Information

**DOI:** 10.3390/e22070741

**Published:** 2020-07-04

**Authors:** Jorge Augusto Karell-Albo, Carlos Miguel Legón-Pérez, Evaristo José Madarro-Capó, Omar Rojas, Guillermo Sosa-Gómez

**Affiliations:** 1 Instituto de Criptografía, Universidad de La Habana, Havana 10400, Cuba; jorgekarellalbo@gmail.com (J.A.K.-A.); clegon58@gmail.com (C.M.L.-P.); ejmcapo@gmail.com (E.J.M.-C.); 2 Facultad de Ciencias Económicas y Empresariales, Universidad Panamericana, Álvaro del Portillo 49, Zapopan, Jalisco 45010, Mexico; orojas@up.edu.mx

**Keywords:** independence, statistical randomness tests, NIST, mutual information

## Abstract

The analysis of independence between statistical randomness tests has had great attention in the literature recently. Dependency detection between statistical randomness tests allows one to discriminate statistical randomness tests that measure similar characteristics, and thus minimize the amount of statistical randomness tests that need to be used. In this work, a method for detecting statistical dependency by using mutual information is proposed. The main advantage of using mutual information is its ability to detect nonlinear correlations, which cannot be detected by the linear correlation coefficient used in previous work. This method analyzes the correlation between the battery tests of the National Institute of Standards and Technology, used as a standard in the evaluation of randomness. The results of the experiments show the existence of statistical dependencies between the tests that have not been previously detected.

## 1. Introduction

Sequences of random numbers are of crucial importance in cryptographic applications, such as prime number generation for RSA encryption and secret keys and initialization vectors in symmetric encryption, but the most common application is in secret key generation [1]. The security of cryptographic applications is based, to a large extent, on the sequences randomness degree [2]. These sequences are obtained by *random number generators* (RNG) [3], which in turn can be classified in two groups according to their randomness source: a generator that needs an input called a seed, and generates an output by algorithmically processing this seed, is called a *pseudo-random number generator* (PRNG), and a generator that uses unpredictable events as a source of randomness is called a *truly random number generator* (TRNG). In most of the software systems that implement cryptography, PRNGs are used [4] due to their easy usability. PRNGs are based on deterministic algorithms, so it is necessary to examine the output to confirm that they are appropriate for cryptographic applications. This output is statistically analyzed using one or more randomness tests, also known as randomness hypothesis tests, and the results are evaluated to determine the randomness of the generator. The statistic associated with each randomness test tries to detect the presence or absence of a certain pattern; if a pattern is detected, the non-randomness of the sequence can be inferred.

There are numerous statistical randomness tests [5,6,7]; some account for more than one hundred such tests [2,8]. The existence of such a large number of tests used to detect different patterns, usually leads to the need to apply more than one test. Thus, one main problem arises: how to select the minimum set of tests, such that the greatest possible number of deviations from randomness is detected. In [5], the application of more than one test is proposed to arrive at reliable results, relying on the strengths of each of the tests used. Any selected subset of tests to evaluate a generator is usually called a *test battery*.

Ideally, different randomness tests should evaluate completely different randomness characteristics. However, the complex nature of randomness deems such an endeavor far from trivial. Two conceptually different tests can be, in essence, evaluating the same characteristic of randomness and therefore producing correlated results, which overestimate the properties of a PRNG. Then, the one important problem is to efficiently determine this correlation. In [9,10], the study of the correlations between statistical randomness tests was proposed using Pearson’s correlation coefficient (PCC); however, it does not detect nonlinear correlations. For this reason, the fundamental objective of this work is to design a new general method by using mutual information to detect linear and non-linear correlations. In this case, the statistical randomness tests present in the test battery of the National Institute of Standards and Technology (NIST) will be evaluated. The intention is to calculate the mutual information between the *p*-values and the statistics of different randomness tests present in the NIST battery. The tests under study are shown in the Table 1.

## 2. Preliminaries

### 2.1. Randomness Assessment

One of the first authors to discuss the randomness of a PRNG was Donald Knuth in his now iconic book *The Art of Computer Programming* [3]. Knuth presented a complete battery of statistical randomness tests; this battery was later adapted and extended by Marsaglia [11]. Marsaglia’s battery called DIEHARD includes implementations of tests in ANSI C language [11]. Among the most cited test batteries, according to [2], are: Knuth Test Suite [12]; DIEHARD Test Suite of Marsaglia [11]; CRYPT-X [13]; TestU01 [14]; the statistical test suite of the NIST [5]. Although some classic tests are presented in Knuth’s test battery, many of these tests can effectively detect non-random sequences. On the other hand, the popular DIEHARD battery requires quite long sequences, and other batteries, such as the so-called CRYPT-X, are commercial products; the well-known NIST battery expanded the study to calculate the quality of different number generators [5], and because it constitutes a standard it was selected for the study in this work.

#### NIST Randomness Tests

Since its emergence, the NIST battery [5] has been widely used due to its standard nature, although it has also received some criticism [4,10,15]. Table 1 presents the 17 statistical randomness tests included in this battery, because the two versions of the CUSUM test (forward and backward), and the two versions of the serial test (1 and 2), are included.

### 2.2. Mutual Information

The *mutual information* (MI) between two variables X,Y∈R, is defined as

$$I\left(X,Y\right)=\int_{Y}\int_{X}p\left(x,y\right)\log\left(\frac{p(x,y)}{p(x)p(y)}\right)dxdy,$$

where p(x,y) is the joint probability function of *X* and *Y*, and p(x) and p(y) are the marginal probability distribution functions of *X* and *Y*, respectively. Mutual information has the following properties: I(X,Y)=I(Y,X); I(X,X)=H(X); I(X,Y)≤min(H(Y),H(X)); I(X,Y)≥0; I(X,Y)=0 if and only if *X* and *Y* are independent. The mutual information between *X* and *Y* variables can be defined in terms of entropy [16] as

I(X,Y)=H(Y)−H(Y∣X)=H(X)+H(Y)−H(X,Y),

where H(X) and H(Y) are the marginal entropy of the variables *X* and *Y*, respectively, H(Y∣X) is the entropy of *Y* conditional on *X*, and H(X,Y) is the joint entropy of both variables [16].

The definition of entropy for a continuous random variable was introduced in 1948 by Shannon [17]. Let *X* be a random vector with values in $R^{d}$ and p(x) its probability density function; then its *differential entropy* is defined by

H(X)=∫p(x)lnp(x)dx,

where ln is the natural logarithm. The discrete case can be defined as follows: let *X* be a random variable which takes values $x_{1},\ldots ,x_{m}$, with each of those values having probability $p_{i}=p\left(x_{i}\right);i=1,\ldots ,m$. The entropy of *X* is then defined as

$$H\left(X\right)=-\sum_{i=1}^{m}p\left(x_{i}\right)\ln p\left(x_{i}\right).$$

The joint entropy H(X,Y) of two discrete random variables *X* and *Y* is defined analogously by

$$H\left(X,Y\right)=-\sum_{i=1}^{m_{X}}\sum_{j=1}^{m_{Y}}p\left(x_{i},y_{j}\right)\ln p\left(x_{i},y_{j}\right),$$

where $p(x_{i},y_{j})$ denotes the joint probability of *X* in state $x_{i}$ and *Y* in state $y_{j}$. If the random variables *X* and *Y* are statistically independent, the joint entropy becomes H(X,Y)=H(X)+H(Y). In general, joint entropy can be expressed in terms of conditional entropy H(X|Y), as H(X,Y)=H(X∣Y)+H(Y), where

$$H\left(X|Y\right)=-\sum_{i=1}^{m_{X}}\sum_{j=1}^{m_{Y}}p\left(x_{i},y_{j}\right)\ln p\left(x_{i}|y_{j}\right),$$

and $p(x_{i}|y_{j})$ denotes conditional probability. When the probability distributions are not known, it is not possible to calculate the exact value of I(X,Y) and it is necessary to calculate an estimator $\hat{I}\left(X;Y\right)$ from a sample [18,19,20].

### 2.3. Problems Associated with Applying too Many Tests

Although we have seen the variety of statistical randomness tests that exist to assess randomness, applying blind tests does not necessarily mean that reliable results are being obtained. Randomness tests are costly processes in terms of time and computational power. To test randomness, one must first generate different sequences and then apply one or more statistical randomness tests to these sequences, to conclude whether the sequence or the PRNG is random or not. Related to this topic, there are two very well-documented problems in the literature: the problem of multiple tests and test independence.

#### Multiple Problem Tests and Independence between Statistical Randomness Tests

The first problem, the one of multiple tests, raises the point that the application of various tests may be affecting the decision in a PRNG. Each test on a battery is applied independently. If all or a predetermined portion of the evidence concludes that the PRNG generates random numbers, it follows that the degree of credibility in the PRNG’s randomness is strong. One might be led to believe that by applying many tests, the ideal PRNG is being selected correctly. In this way, although it sounds logical, problems of multiple tests are being generated. Demirhan and Bitirim, in [2], state that

$$P\left(fail\;at\;least\;onetest\right)=1-P\left(not\;fail\;any\;test\right)=1-{\left(1-\alpha \right)}^{k},$$

where *k* is the number of tests applied and α the level of significance. For example, if k=5 tests are applied, with a significance level of 0.05, one has that $1-{\left(1-0.05\right)}^{5}\approx 0.23$. Then there is a 23% chance of deciding that sequences generated by an RNG are non-random in at least one test, even when all tests indicate that the sequence is random. It can be noted that when more than one test is used simultaneously to evaluate the randomness of a PRNG, the probability of rejecting the null hypothesis increases with the increase in the number of *k* tests; i.e., one could be rejecting a generator, when it actually generates random numbers.

The second problem, the one of test independence, was addressed by Soto [21] in 1999, who stated that the tests included in a battery must be independent. Therefore, they must be chosen in such a way that they are not correlated with each other. Then a good selection can increase the efficiency of a battery. In 2008, Turan [22] studied the independence of randomness tests and their effect on the correct functioning of batteries. Two tests *T*_1_ and *T*_2_ are considered *independent* if the distributions of their statistical randomness tests (and the corresponding *p*-values) are independent; i.e, $p\left(t_{1}|t_{2}\right)=p\left(t_{1}\right)$ and $p\left(t_{2}|t_{1}\right)=p\left(t_{2}\right).$

One way to avoid both problems is to reduce the number of statistical randomness tests that are applied, selecting the appropriate tests in such a way that two different tests that evaluate the same characteristic of randomness are not applied. This can be formalized by detecting correlated statistical randomness tests on different batteries.

## 3. Some Previous Results

There are many works on the evaluation of the correlation between statistical randomness tests; see [9,10,15,23,24]. In this work, the correlation between the statistical randomness tests of the NIST battery will be evaluated, so only some reported results on the correlation between these tests will be described in order to be able to make comparisons with respect to the results obtained. In [22], it was stated that tests that measured similar properties should not be included in the same battery; therefore, one of each class should be chosen.

As an alternative to the definition of independence, Turan considered the independence of two tests $T_{1}$ and $T_{2}$, such that their rejection regions were independent of the selection of α. He also introduced the concept of sensitivity to analyze the correlation from the results of the tests in transformed sequences. According to [22], if the transformation significantly changes the *p*-values, then the composition of the transformation and the test should be included to increase battery coverage. As transformations, the complement, *l*-displacement, change of the *i*-th bit, inversion, and the *l*-th derivative were chosen. As a result of their study, it was found that the statistical randomness tests frequency, overlapping template, the longest run of ones, random walk height, and maximum order complexity, producing correlated results when using short sequences (20 and 30 bits). Although the Turan studies open up a new approach when considering transformed sequences, it is important to note that it uses short sequences and these detected correlations decrease as the number of input bits increases; i.e., when the length of the sequence increases. For this reason, we do not classify these results as significant for our work.

Doganaksoy in 2008 [9] studied the dependencies of some NIST tests, taking into account their corresponding *p*-values and analyzed the sensitivity of these tests under different transformations. He generated $10^{5}$ sequences of 5000 bits each and applied them as basic transformations of inversion, complement, 1-displacement, and 8-displacement. To measure the correlation, Doganaksoy used Pearson’s correlation coefficient

$$r_{xy}=\frac{cov(X,Y)}{\sigma (x)\sigma (y)}=\frac{\sum x_{i}y_{i}-\sum x_{i}\sum y_{i}}{\sqrt{n\sum x_{i}^{2}-{\left(\sum x_{i}\right)}^{2}}\sqrt{n\sum y_{i}^{2}-{\left(\sum y_{i}\right)}^{2}}},$$

where $x_{i}$ and $y_{i}$ are the *i*-th blocks of the sequence for which the correlation is being investigated, and *n* is the number of *p*-values. As a result, Doganaksoy detected the existence of a correlation between the tests shown in Table 2.

It is important to take into account that Pearson’s correlation coefficient only detects the degree of linear correlation between the *p*-values. The closer the coefficient is to 1, the stronger the correlation between the tests. If two tests are independent, the correlation coefficient is 0, but the reciprocal is generally not true. If the correlation coefficient is 0, this only means that there is no linear relationship between the tests.

In [15], a new general method based on the different distribution functions of *p*-values was introduced to estimate the correlation between two statistical randomness tests. The reasoning on which the new method is based starts by assuming the independence of the evidence. If two tests are independent, the results of one of them should not influence the other. Two different tests are denoted as $T_{X}$ and $T_{Y}$. The distribution of *p*-values for a random dataset tested by $T_{X}$ is *X* and its probability density is $f_{x}$. The distribution of *p*-values for a random dataset tested by $T_{Y}$ is *Y* and its probability density is $f_{y}$. Then, as $T_{X}$ and $T_{Y}$ are independent, the random variables *X* and *Y* are independent; then the distribution of Z=X−Y is deduced. The probability density of Z=X−Y=X+W results in

$$f\left(z\right)=f_{X}\cdot f_{W}=\int_{-\infty }^{\infty }f_{X}\left(x\right)\cdot f_{W}\left(z-x\right)dx,$$

where W=−Y. Then,

$$f\left(z\right)=\left\{\begin{array}{cc} 0 & z<-1\\ z+1 & -1\leq z<0\\ -z+1 & 0\leq z\leq \;1\\ 0 & z>1 \end{array}\right.$$

Therefore, if the two tests are independent, the distribution of the difference of the *p*-values must follow the distribution with probability density function f(z). In [15], G-DES, G-BBS, G-ANSI, and G-SHA-1, were used as generators; 100 sets were generated for each generator and each set contained 300 binary sequences of 1,000,000 bits. The results detected the correlations shown in Table 3.

In [10], studies on the correlations between tests were carried out using Pearson’s correlation coefficient, but this time he divided the tests into two groups: the tests that can be applied to short sequences and those that can be applied to long sequences. The experiments generated 200,000 sequences of $2^{10}$ bits considered short, and 200 sequences of $2^{20}$ bits considered long, always bearing in mind that the tests that can be applied to short sequences, can also be applied to long sequences, but not the other way around. The behavior of the tests in transformed sequences was also analyzed. If two tests react similarly to transformations, then these tests are said to be *structurally similar*. Transformations included inversion, binary derivative, *l*-Offset, *i*-th bit change, bit masking, and bit flip. In this case, the results detected the correlations shown in Table 4.

## 4. The Proposed Method to Detect the Correlation between Statistical Randomness Tests

To detect the correlation between statistical randomness tests, the joint application of two tests is modeled as a non-symmetric binary channel, such that if two tests are correlated, then the mutual information of the channel will be different from zero and an increasing function of the degree of correlation between tests. The main advantage of this method is expected to be the ability of mutual information to detect other types of functional dependency. A possible disadvantage may be due to the influence of the estimator of mutual information on the precision of the results; therefore, the estimator must be chosen carefully. The proposed method is described in the following steps:

Step 1. Select the random number generators.
  - The generators used must generate outputs that satisfy the random conditions.
Step 2. Build the data samples using the selected generators.
  - Generate *n* sequences of random numbers of length *L* to evaluate them using the selected statistical tests.
Step 3. Evaluate each of the *n* sequences by the *k* statistical tests and obtain their corresponding *n* *p*-values, or test statistics, $p_{i}^{e}$ for each test $T_{i}$ (with i=1,…,k and e=1,…,n).
Step 4. Calculate the mutual information between the sequences of *p*-values, or test statistics, to detect the presence of correlations.
  - Estimate the MI between all the pairs $\left(T_{i},T_{j}\right)$ of sequences of *p*-values or test statistics, to detect the presence of correlation, using the MI expression based on entropy *H* and some estimator of *H* with low mean squared error (MSE), over the number *n* of sequences generated in step 2.
Step 5. Determine the significant correlations to conclude the correlation between the tests. The MI values were grouped by property 1 of the MI in the triangular matrix M:
  $$M={\left(\begin{array}{cccc} I(T_{1},T_{1}) & I(T_{1},T_{2}) & \ldots & I(T_{1},T_{k})\\ 0 & I(T_{2},T_{2}) & \ldots & I(T_{2},T_{k})\\ \vdots & \vdots & \ddots & \vdots \\ 0 & 0 & \ldots & I(T_{k},T_{k}) \end{array}\right)}_{k\times k}$$
  where $I(T_{i},T_{j})$ represents the MI between the *i* and *j*. Taking into account property 2 we have
  $$M={\left(\begin{array}{cccc} H(T_{1}) & I(T_{1},T_{2}) & \ldots & I(T_{1},T_{k})\\ 0 & H(T_{2}) & \ldots & I(T_{2},T_{k})\\ \vdots & \vdots & \ddots & \vdots \\ 0 & 0 & \ldots & H(T_{k}) \end{array}\right)}_{k\times k}$$
  where $H(T_{i})$ represents the entropy of the variable $T_{i}$. The resulting matrix is a triangular matrix where the diagonal contains $H(T_{i})$.

For a better interpretation, we proceeded to normalize the MI values representing by $I^{\prime }\left(T_{i},T_{j}\right)$. There are three main variants to normalize mutual information between two variables $T_{i}$ and $T_{j}$ [25]: dividing by the maximum entropy, $\max\{H\left(T_{i}\right),H\left(T_{j}\right)\}$, dividing by the minimum entropy, $\min\{H\left(T_{i}\right),H\left(T_{j}\right)\}$ and dividing by the mean of the two entropies $\left[H\left(T_{i}\right)+H\left(T_{j}\right)\right]/2$. In [25] it is argued and recommended to divide between the minimum entropy. If $T_{i}$ and $T_{j}$ have the same distribution, their entropies are equal $H\left(T_{i}\right)=H\left(T_{j}\right)$ and the three variants coincide and are equal to the ρ coefficient proposed in [16]:

$$I^{\prime }\left(T_{i},T_{j}\right)=\rho =\frac{I(T_{i},T_{j})}{H(T_{i})}.$$

The case of this work, $T_{i}$ and $T_{j}$ have the same distribution because the sequences were randomly constructed by step one, and therefore the *p*-values distribute uniformly [5]. Then

$$M={\left(\begin{array}{cccc} 1 & I^{\prime }\left(T_{1},T_{2}\right) & \ldots & I^{\prime }\left(T_{1},T_{k}\right)\\ 0 & 1 & \ldots & I^{\prime }\left(T_{2},T_{k}\right)\\ \vdots & \vdots & \ddots & \vdots \\ 0 & 0 & \ldots & 1 \end{array}\right)}_{k\times k}$$

where $I^{\prime }\left(T_{i},T_{j}\right)$ is the value of the normalized mutual information (NMI) between the random variables $T_{i}$ and $T_{j}$ given an estimator of the mutual information.

The procedure for calculating the normalized mutual information matrix can be summarized in Algorithm 1.
**Algorithm 1:** Normalized mutual information matrix.
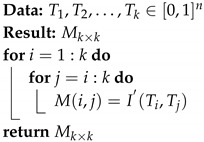


To decide whether the value $I^{\prime }\left(T_{i},T_{j}\right)$ is significantly greater than 0, and thus conclude whether there is any kind of dependency between both variables, the following hypothesis was formulated,

$$\begin{array}{cc} H_{0} & :I^{\prime }\left(T_{i},T_{j}\right)=0\\ H_{1} & :I^{\prime }\left(T_{i},T_{j}\right)>0 \end{array}$$

where $H_{0}$ is the null hypothesis that raises independence and $H_{1}$ is the alternative hypothesis, where there would be some kind of association between $T_{i}$ and $T_{j}$. From a sample $\left(p_{i}^{1},p_{j}^{1}\right),\ldots ,\left(p_{i}^{n},p_{j}^{n}\right)$, it must be decided whether to reject the null hypothesis $H_{0}$ of independence between $T_{i}$ and $T_{j}$; i.e., if $I^{\prime }\left(T_{i},T_{j}\right)$ is significantly greater than zero then the null hypothesis $H_{0}$ is rejected. For this it is necessary to calculate a *p*-value associated with the test and if it is less than the selected significance level, then there will be sufficient evidence against $H_{0}$.

Since the distribution of the MI under $H_{0}$ is not known, a permutations test [26,27] will be performed.

*Permutation test*

1. Construct the permuted samples $\left(T_{i},\pi_{u}\left(T_{j}\right)\right),\forall u=\left\{1,\ldots ,q\right\}$ with *q* the number of permutations used, in such a way that the possible association between $T_{i}$ and $T_{j}$ disappears, with π being the permutation *u* of the elements of $T_{j}$, that is to say,
  - $\pi_{u}\in S_{n},\forall u=\left\{1,\ldots ,q\right\}$.
  - $\pi_{u}\neq \pi_{v}$, for u≠v.
  - $\pi_{0}$ is the identity of $S_{n}$.
2. Estimate the MI of the permuted samples, to obtain ${\left\{Z_{u}\right\}}_{u=0}^{q}$, where $Z_{u}=I^{\prime }\left(T_{i},\pi_{u}\left(T_{j}\right)\right)$.
3. The *p*-value associated with the test of the form is calculated
  (1) $$p-value=\frac{\sum_{u=1}^{q}1_{\geq Z_{0}}\left(Z_{u}\right)}{q},$$
  where $1_{\geq Z_{0}}\left(Z_{u}\right)$ is the defined indicator function of the form
  (2) $$1_{\geq Z_{0}}\left(x\right)=\left\{\begin{array}{ccc} 1 & if & x\geq Z_{0}\\ 0 & if & x<Z_{0} \end{array}\right.$$
4. If p−value≥α, then the null hypothesis is accepted. If p−value≤α, then the null hypothesis is rejected.

## 5. Correlation Assessment of NIST Tests

The intention is to calculate the mutual information between the *p*-values of the NIST randomness tests so that it can be compared with previous results. Data samples were constructed using different PRNGs implemented in the NIST battery (SHA-1, Linear Congruential, Micali–Schnorr, Blum-Blum-Shub). The experiments were carried out in the same way for each generator: n=10,000 sequences of length L=1,000,000 bits were generated, with q=10,000 permutations and a significance level α=0.001. The results obtained are similar for all PRNGs.

### 5.1. Selection of the Number of Sequences and MI Estimator

**Figure 1:**
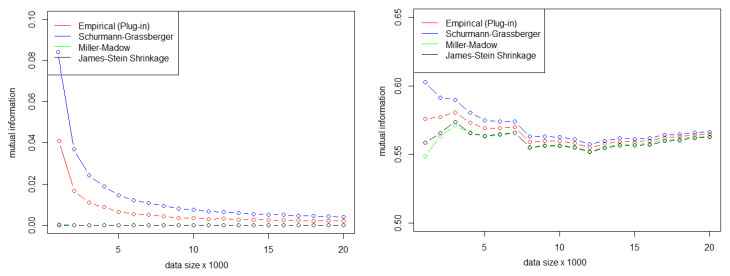
Estimation of mutual information for different samples sizes.

To select the number of sequences and the estimator of the mutual information, MI was calculed for different samples sizes and certain estimators. The mutual information was estimated from its expression in terms of entropy. The estimation was made for the two possible cases, correlated variables and independent variables. The pairs of random variables were selected from the *p*-values of the previously studied test [10,15]. The behavior of the estimated MI values was then analyzed, as the number of observations of each random variable increased. Among the entropy estimators analyzed, the ones in the infotheo package [28] were chosen. These include the plug-in (emp), the Miller–Madow correction (mm), James–Stein shrinkage (shrink), and the Schurmann–Grassberger estimator (sg). Figure 1 shows how the estimated values of the MI approach as the number of observations increases.

In Table 5 and Table 6, it can be seen that the maximum difference between the estimated values decreases considerably when the observations increase in the case of independent variables.

In Table 5, for more than 10,000 observations the maximum difference is 0.0074 and for correlated variables in Table 6, it is 0.0065.

Taking into account that in our problem the number of observations equals the number of sequences analyzed, we consider that if at least 10,000 sequences of pseudo-random numbers are analyzed, the maximum difference between the estimators is very small. However, it is important to note that the Miller–Madow and James–Stein shrinkage estimators for the case of a couple of independent random variable, mutual information takes a value of zero even for small sample sizes. Based on the results obtained, and on those reported in the literature [29,30] on the comparison between these estimators, we decided to use the shrinkage estimator for the experiments.

### 5.2. Sample Discretization

In this paper, we dealt with *p*-values, which are continuous data, so it is necessary to discretize the sample to estimate the entropy of the data. It is known that the discretization method used influences the results of the calculation of mutual information. One of the main problems with different discretization methods is determining the appropriate number of intervals to get good results. Although the choice of the number of intervals is crucial for the discretization process, there is no definitive strategy to determine the optimal number of intervals; therefore, there are several approaches proposed [31]. The chosen discretization method is to divide the domain into K=10 intervals of equal size, which constitutes a simple and fast algorithm.

### 5.3. Correlation between p-Values

In practice, statistical randomness tests do not provide the same results for all possible input parameters. Therefore, it is important to know the restrictions of each test for the selection of these parameters. Table 7 shows the parameters used in each of the 17 statistical randomness tests.

**Figure 2:**
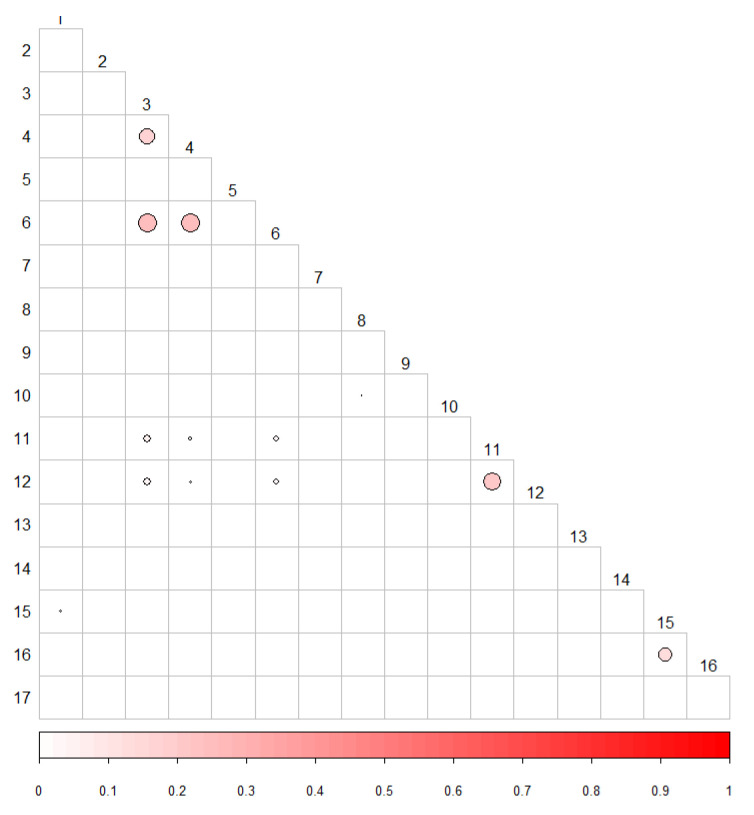
Correlation matrix graph for α=0.001.

Representing the NMI values in Figure 2, we can see which tests have the highest NMI values. The size of the circles and the intensity of the color correspond to the value of the NMI.

This indicates the presence of significantly higher NMI values than would be expected for a couple of independent tests. Table 8 shows the correlations detected.

#### Discussion of Correlations between *p*-Values

**Figure 3:**
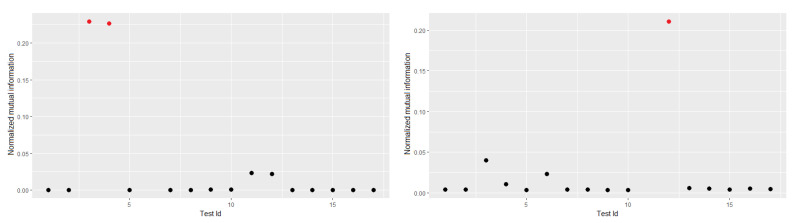
Relationship between tests.

To validate these results, the NMI values can be analyzed between several of the tests. It is evident that the MI values between the Frequency tests and the CUSUM forward and CUSUM backward tests are very high concerning the values of the other tests (Figure 3a). Something similar happens with the random excursions and random excursions variant tests (Figure 3b).

**Figure 4:**
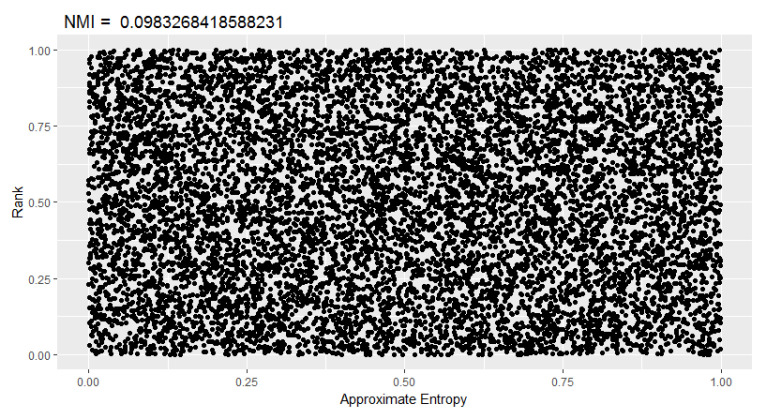
Dispersion of p-values of *approximate entropy* and *rank*.

It can be seen that the normalized mutual information between the random excursions test and the random excursions variant test is high concerning the rest. Other evidence could be the behavior of *p*-values. If the *p*-values of the approximate entropy and rank tests (Figure 4), which did not show evidence of correlation by the NMI, are plotted, no behavior that points towards some type of functional dependency is visible.

**Figure 5:**
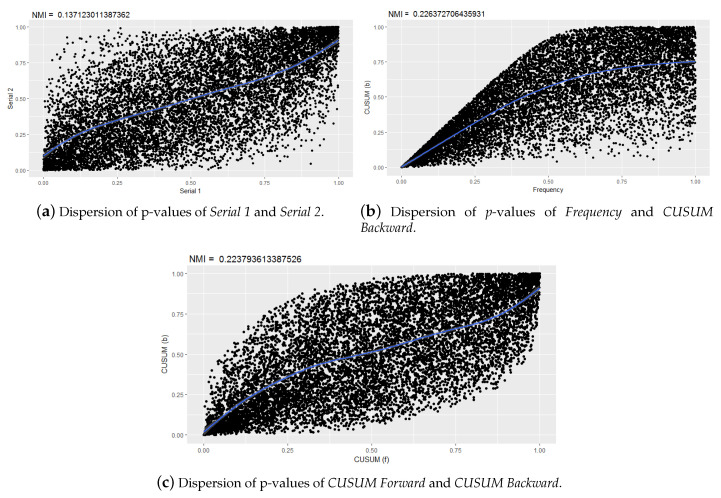
Dispersion of normalized mutual information (NMI) values of various tests.

On the other hand, if we analyze the scatter plots of other pairs of tests, it is observed that as the NMI values increase, the sequences of *p*-values reflect behaviors associated with functional dependencies. This is evidenced by the Serial 1 and Serial 2 tests (Figure 5a), frequency and CUSUM backward (Figure 5b), CUSUM forward, and CUSUM backward (Figure 5c).

### 5.4. Correlation between Statistics

Previous studies on the correlation between statistical randomness tests [9,15] used *p*-values to analyze the correlation. This approach has the advantage of using the same scale and interpretation for the variables studied since they are values between 0 and 1; however, the potentially important information implicit in the statistics is lost. In this section, the approach proposed in [32] is applied, where the correlation between the statistical randomness tests is calculated, using the values of the statistic, instead of the *p*-values. The main advantage is expected to increase the strength of the analysis as more information is exploited.

**Figure 6:**
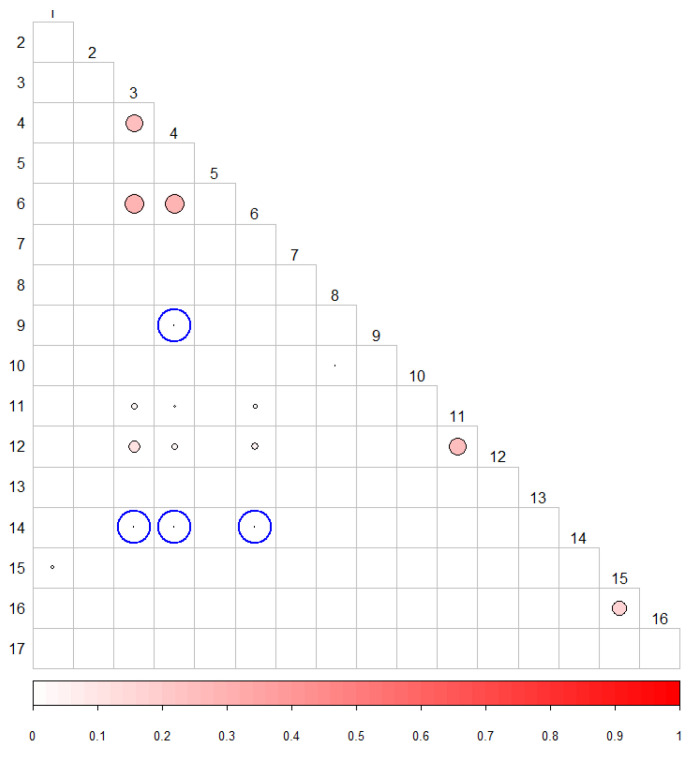
Correlation matrix between the statistics for α=0.001.

For the analysis of the correlation, the same design was used as in the previous experiments, substituting only the *p*-values for the sequences of statistics corresponding to each of the tests. As expected, with this new approach the same correlations are detected as if the *p*-values are used, and new ones appear since information is not lost when calculating the NMI directly from the statistics. The new correlations detected have been emphasized in Table 9, and the correlation matrix with these new circled detected correlations are shown in Figure 6.

#### Discussion of the Correlations between the Statistics

**Figure 7:**
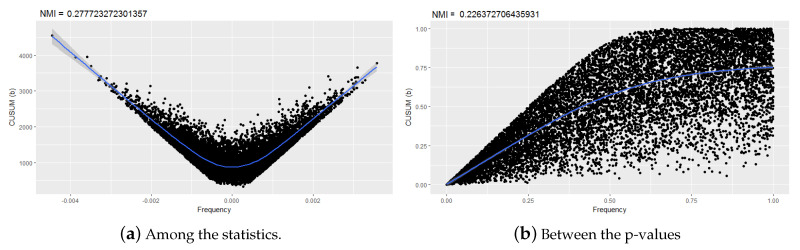
Comparison of data dispersion.

Importantly, MI is able to detect correlated tests if *p*-values are used or if statistics are used, even though the type of functional dependency may change. Figure 7 shows the scatter plots between the frequency and CUSUM backward tests, first among the test statistics, and then among the *p*-values. These results show that the method for calculating the correlation between statistical randomness tests is more effective if the values of the test statistics are used. In this way, this method is capable of detecting new correlations, even the values of NMI of the known correlations increase.

Note in Figure 7a that the dependency has a parabolic shape and cannot be detected by the linear correlation coefficient, but by the normalized mutual information.

### 5.5. Comparison of Results with Previous Work

To make an objective comparison between the results achieved by other methods and ours, it is necessary to fully know the work environment in which they were developed. As initial parameters, it is necessary to know the number of sequences that each method needs to achieve these results and then to know the parameters used by each of the tests, since when these parameters vary, the focus of the test changes significantly and with it the results. The initial parameters used in the different methods are presented in Table 10.

The number *n* of sequences used by the proposed method is reduced by the method presented in [15] and in one of the datasets of [10]. In this work, sequences as long as those of [10,15] were used, which provides reliability to the values of the obtained *p*-values and statistics, and a sample size of 10,000 sequences to guarantee the precision of the estimator of the MI. It is not possible to make a reasonable comparison between our method and the one proposed in [15] regarding the correlations detected, since the publication of the latter does not expose the parameters used for the application of the tests. However, for the two cases analyzed above, it was observed that our method detects more correlations between the statistical randomness tests. If one wants to make a comparison with the results of the different methods, it is also necessary to apply the tests under the same parameters. In the previous methods, the tests that varied their parameters with respect to those predefined by NIST are represented in Table 11.

When applying the proposed method with the parameters of the previous studies, the results shown in Table 12 were obtained. The new correlations have been emphasized; it is evident that our method detects the same correlations as the previous ones and new ones not previously studied.

In Table 12; the first column corresponds to the reference tests to illustrate the correlations; in the following two columns are the results reported in the corresponding bibliographies with respect to each test in the first column; and in the fourth column the results obtained in this work.

**Figure 8:**
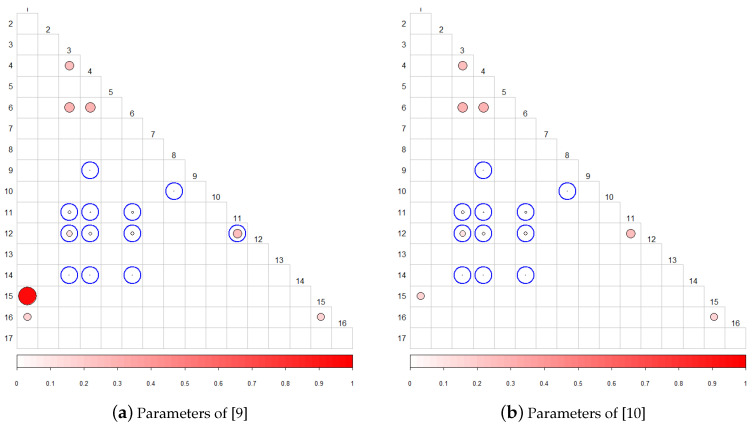
Correlations detected for the parameters used in previous works.

**Figure 9:**
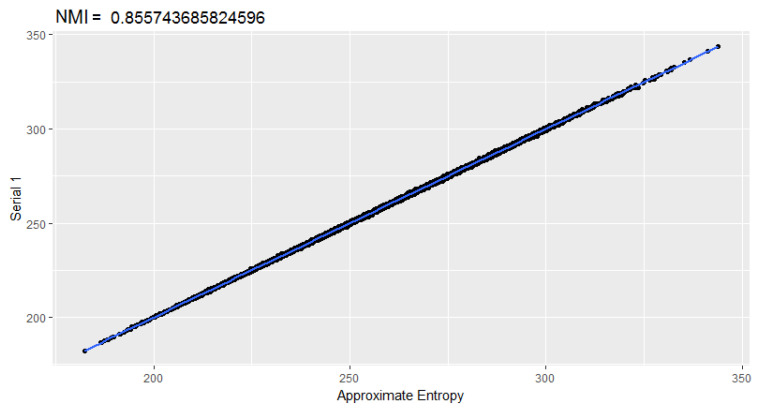
Dispersion between the Approximate entropy and Serial 1 tests.

Figure 8 shows the correlation matrices with the tests detected using the same parameters used in the background. As a substantial revelation, the Approximate entropy test with m=8 is equivalent to the Serial 1 test with m=9 (parameters of [9]); see Figure 9. Also, Serial 2 test is correlated with the approximate entropy test.

This result suggests that the method designed to detect correlation can also be used to suggest options in the selection of test parameters.

## 6. Conclusions

In this work, a method based on mutual information was designed to analyze the correlation between statistical randomness tests. Specifically, the tests present in the NIST battery were analyzed, which constitutes the standard to be used for the analysis of randomness. At first, the *p*-values of the tests were analyzed, and then test statistics were used, which provided better results. Thanks to the properties of the MI, the proposed method is capable of detecting any type of correlation, regardless of its functional dependence, and in this way, we report new correlations not detected in previous works. The results obtained show that it is possible to reduce the number of tests present in a battery, eliminating any redundant tests.

To decide which tests to remove from the battery, it is convenient to analyze the correlation between more than two tests. The analysis of three or more tests does not modify the correlations already detected between pairs of them, but could modify the decision on which tests should be eliminated. The results presented here are the first step in the analysis of deeper correlations between a set of more than two tests.

About the proposed method, some problems arise that raise several future prospects. Among these should be a study going in-depth into the different discretization methods, both supervised and unsupervised, as they could offer improvements to the designed method. Compare this with other decision methods in terms of what values of mutual information are significant. Carry out an extensive analysis of statistical randomness tests available in the literature, without limiting them to a specific battery. On the other hand, it would be interesting to work on detecting the correlation between three or more statistical randomness tests. 

## Figures and Tables

**Table 1:** NIST battery of randomness tests.

Randomness Test	Id	Randomness Test	Id
Approximate Entropy	1	Overlapping Template	10
Block Frequency	2	Random Excursions	11
CUSUM (f)	3	Random Excursions Variant	12
CUSUM (b)	4	Rank	13
FFT	5	Runs	14
Frequency	6	Serial 1	15
Linear Complexity	7	Serial 2	16
Longest Run	8	Universal	17
Non Overlapping Template	9		

**Table 2:** Correlations detected in [9].

Randomness Tests	Related Tests
Frequency	CUSUM(f, b)
CUSUM(f)	CUSUM(b)
Serial 1	Serial 2
Approximate Entropy	Serial(1,2)

**Table 3:** Correlations detected in [15].

Randomness Tests	Related Tests
Frequency	CUSUM, Approximate Entropy
Runs	Approximate Entropy, Serial
Approximate Entropy	Frequency, CUSUM, Runs, Serial
Serial	Runs, Approximate Entropy

**Table 4:** Correlations detected in [10].

Randomness Tests	Related Tests
Frequency	CUSUM (b, f)
CUSUM (b)	CUSUM (f)
Serial 1	Serial 2
Approximate Entropy	Serial(1,2)
Random Excursion	Random Excursion Variant

**Table 5:** Value differences for independent variables.

est/obs	1000	5000	10,000	15,000	20,000
emp	0.041	0.0064	0.0034	0.0025	0.002
mm	0.0005	0	0	0	0
shrink	0	0	0	0	0
sg	0.0806	0.0145	0.0074	0.0052	0.004
max. Dif.	0.0806	0.0145	0.0074	0.0052	0.004

**Table 6:** Value differences for correlated variables.

est/obs	1000	5000	10,000	15,000	20,000
emp	0.5761	0.5691	0.5597	0.5592	0.5647
mm	0.5486	0.5632	0.5567	0.5574	0.5632
shrink	0.5585	0.5636	0.5561	0.5565	0.5625
sg	0.6027	0.5749	0.5626	0.5612	0.5662
max. Dif.	0.0541	0.0117	0.0065	0.0047	0.0037

**Table 7:** Parameters used for the tests.

Randomness Test	Parameter	Value
Approximate Entropy	m: first block size	10
Block Frequency	M: block size	128
Linear Complexity	M: block size	500
Longest Run	M: block size	128
Non Overlapping Template	template	000000001
Overlapping Template	template	111111111
Random Excursions	internal state	x=−4
Random Excursions Variant	internal state	x=−9
Rank	M: rows of each matrix	32
	Q: columns of each matrix	32
Serial	m: block size	16
Universal	L: block size	7
	Q: initialization blocks	10

**Table 8:** Correlated tests for α=0.001.

Random Test	Related Test
Approximate Entropy	Serial 1
CUSUM (f)	CUSUM (b), Frequency, Random Ex., Random Ex. Variant
CUSUM (b)	Frequency, Random Ex., Random Ex. Variant
Frequency	Random Ex., Random Ex. Variant
Longest Run	Overlapping Template
Random Excursions	Random Excursions Variant
Serial 1	Serial 2

**Table 9:** New correlation detected with the proposed method using statistics.

Randomness test	Related Test
Approximate Entropy	Serial 1
CUSUM (f)	CUSUM (b), Frequency, Random Ex., Random Ex. Variant
CUSUM (b)	Frequency, Random Ex., Random Ex. Variant
Frequency	Random Ex., Random Ex. Variant
Longest Run	Overlapping Template
Random Excursions	Random Excursions Variant
Serial 1	Serial 2
*Runs*	*CUSUM (f, b), Frequency*
*Non Overlapping Template*	*CUSUM (b)*

**Table 10:** Initial parameters used in previous works.

Method	Number *n* of Sequence	Sequence Length *L* (bits)	Metric Used
[9]	100,000	5000	Pearson
[15]	300	1,000,000	Setting to distance of Fan [15]
[10]	200,000	1024	Pearson
	200	1,048,576	Pearson
Proposed method	10,000	1,000,000	MI

**Table 11:** Parameters of the tests in previous works.

Method	Approximate Entropy	Serial
[9]	8	9
[10]	14	16
predefined by NIST	10	16

**Table 12:** Comparison of the correlation of evidence obtained with previous results [9,10].

Randomness Tests	Correlated Tests		
	[9]	[10]	Proposed Method
Frecuency	CUSUM(f, b)	CUSUM(f, b)	CUSUM(f, b) *Random Ex.* *Random Ex. Variant* *Runs*
App. Entropy	Serial(1,2)	Serial(1,2)	Serial 1
CUSUM(f)	CUSUM(b)	CUSUM(b)	CUSUM(b) *Random Ex.* *Random Ex. Variant* *Runs*
*CUSUM(b)*	CUSUM(f)	CUSUM(f)	CUSUM(f) *Non Overlapp.* *Random Ex.* *Random Ex. Variant* *Runs*
Serial 1	Serial 2	Serial 2	Serial 2
Random Ex.		Random Ex. Variant	Random Ex. Variant
*Longest*			*Overlapp.*
